# Physicochemical and Biocompatibility Properties of Type I Collagen from the Skin of Nile Tilapia (*Oreochromis niloticus*) for Biomedical Applications

**DOI:** 10.3390/md17030137

**Published:** 2019-02-26

**Authors:** Wen-Kui Song, Dan Liu, Lei-Lei Sun, Ba-Fang Li, Hu Hou

**Affiliations:** 1 College of Food Science and Engineering, Ocean University of China, No.5, Yu Shan Road, Qingdao 266003, China; sprdice@outlook.com; 2 College of Chemistry and Chemical Engineering, Ocean University of China, 238 Songling Road, Qingdao 266003, China; liudan090@163.com; 3 College of Life Science, Yantai University, Yantai 264005, China; leilei.198966@163.com

**Keywords:** Nile tilapia collagen, biomedical application, characterization

## Abstract

The aim of this study is to investigate the physicochemical properties, biosafety, and biocompatibility of the collagen extract from the skin of Nile tilapia, and evaluate its use as a potential material for biomedical applications. Two extraction methods were used to obtain acid-soluble collagen (ASC) and pepsin-soluble collagen (PSC) from tilapia skin. Amino acid composition, FTIR, and SDS-PAGE results showed that ASC and PSC were type I collagen. The molecular form of ASC and PSC is (α_1_)_2_α_2_. The FTIR spectra of ASC and PSC were similar, and the characteristic peaks corresponding to amide A, amide B, amide I, amide II, and amide III were 3323 cm^−1^, 2931 cm^−1^, 1677 cm^−1^, 1546 cm^−1^, and 1242 cm^−1^, respectively. Denaturation temperatures (Td) were 36.1 °C and 34.4 °C, respectively. SEM images showed the loose and porous structure of collagen, indicting its physical foundation for use in applications of biomedical materials. Negative results were obtained in an endotoxin test. Proliferation rates of osteoblastic (MC3T3E1) cells and fibroblast (L929) cells from mouse and human umbilical vein endothelial cells (HUVEC) were increased in the collagen-treated group compared with the controls. Furthermore, the acute systemic toxicity test showed no acute systemic toxicity of the ASC and PSC collagen sponges. These findings indicated that the collagen from Nile tilapia skin is highly biocompatible in nature and could be used as a suitable biomedical material.

## 1. Introduction

Collagen is the main structural protein in the extracellular matrix (ECM), constituting approximately 30% of the whole body protein content in animals [1]. More than 29 different types of collagen have been identified and described. In the human body, most of the collagen is type I [2]. The collagen protein contains triple-helix structures that consist of three almost identical polypeptide chains [3]. Type I collagen is present in bone, skin, dentin, cornea, blood vessels, fibrocartilage, and tendon; it has the unique ability to form fibrils that have high tensile strength and important functions [4,5]. In the past decades, it has been widely used in food manufacturing and the cosmetics industry [6].

Recently, the interest in collagen has become widespread among medicine and tissue engineering because of its predominance in the ECM, excellent biocompatibility, low antigenicity and available methods of isolation from a variety of sources [7,8]. Currently, the major sources of collagen are the tendon or skin of bovine and porcine. However, the prevalence of transferring diseases including foot-and-mouth disease (FMD), bovine spongiform encephalopathy (BSE), and transmissible spongiform encephalopathy (TSE), and the religious barriers of Muslims and Jews [9,10,11], have limited its application [12]. Therefore, it is essential to find a safety source of collagen for human application. Marine collagen has been isolated and characterized from various marine sources, and can generally be categorized according to source: vertebrates or invertebrates. Vertebrates sources include cat fish [13], silvertip shark [14], salmon [15], yellow tuna [16], and marine mammals such as minke whale [17]. Invertebrates source include jellyfish [18,19,20], squid [21], and sponges [22,23,24,25]. Researchers have demonstrated that similar characteristics exist between marine collagen and mammalian [26,27,28]. However, some differences exist between collagen extracted from marine sources and collagen extracted from mammals. Compared with the mammalian collagen, marine collagen has lower gelling and melting temperatures, but relatively higher viscosities than equivalent bovine forms [29]. Fish collagens show a similar amino acid distribution to mammalian collagen, with decreased amounts of proline and hydroxyproline, and increased serine, threonine, and in some cases, methionine and hydroxylysine [30]. Compared with mammalian collagen, the difference in the amino acid distribution of fish collagen causes labile cross-links and heat sensitivity [31]. In recent years, marine collagen has been widely used in medicine and tissue engineering fields [32], such as cartilage [33], corneal [34], ligament [35], muscle [36], skin [37], tracheal [38], and vascular [39].

The Nile tilapia (*Oreochromis niloticus*) is a worldwide cultured fish that possesses an important position in China’s aquaculture and exports industry [26]. Tilapia skin is a main by-product of its processing, which contains approximately 30% collagen [40]. Our previous study revealed that both acid-soluble collagen (ASC) and pepsin-soluble collagen (PSC) extracted from Nile tilapia skin can be used as raw materials in food and cosmetic preparation [41]. Further, we want to explore whether either is suitable for use in biomedical applications. However, the biocompatibilities of pure collagen extracted from Nile tilapia need be addressed, since the biocompatibilities of fish collagen are profoundly influenced by the molecular composition and arrangement, which is thought to be varied by different extraction methods. In this work, we extract acid-soluble (ASC) and pepsin-soluble (PSC) collagen from Nile tilapia skin, and then describe their physical properties, chemical properties, and biocompatibilities, in order to explore the possibility for applications in biomedical fields.

## 2. Results and Discussion

### 2.1. Preparation of ASC and PSC

The yields (dry weight basis, 19.07% and 19.61% respectively) of ASC and PSC were in highly agreement with former reports [42,43]. Comparison of the collagens extract from different fish have been reported, including the skin of paper nautilus (55.2%) [44], bigeye snapper (1.59%) [45], deep-sea redfish (10.3%) [46], Japanese sea bass (40.7%) [47], yellow sea bream (40.1%) [48], and grass carp (45.3%) [43]. In contrast, the yield of collagen from tilapia was different from these reported species. The results suggest that some discrepancies might exist among these fish species. In addition, the yield of ASC was a little bit lower than the yield of PSC, this might be due to the disadvantage of the solubility of cross-links formed through the reaction of aldehyde with lysine and hydroxylysine at telopeptide helical sites [49], which also can be explained by the results of Fourier transform infrared (FTIR) analysis. Besides the effects on solubility, the pepsin that is used in the extraction of PSC might bring other changes, such as the stability and biocompatibility of the resultant collagens.

### 2.2. Amino Acid Composition

Table 1 shows the amino acid composition of ASC and PSC, which is expressed as residues per 1000 total amino acid residues. According to Table 1, glycine is the most important component in ASC and PSC, with 322 and 343 residues, accounting for about one-third of the total amino acid residues. It is slightly higher than common aquatic organisms carp skin (311 residues), cod skin (308 residues), squid skin (269 residues) [50,51], and very similar to land mammals’ bovine skin (320 residues), bovine skin (334 residues), and porcine skin (326 residues) [52,53]. Glycine is the most important amino acid in collagen. All members of the collagen family have a tripeptide (Gly-X-Y) repetitive structure, which plays an important role in the formation of the triple-helix structure. The tripeptides (Gly-X-Y) are repeatedly arranged on each chain of collagen, accounting for about 20–30% of all tripeptide structures. The X position of Gly-X-Y is often occupied by proline, which is consistent with the result in Table 1 (115 and 106 proline residues in ASC and PSC).

The existence of the triple-helix structure is the most direct evidence to distinguish collagen from gelatin. [54]. As is known, electrospinning is a method of stretching a polymer solution to fibers that have a diameter of about several hundred nanometers by electrostatic force. Due to its wide applicability, high efficiency, and simplicity, electrospinning is widely used in the field of tissue engineering scaffold materials.

In addition, it is worth noting that the content of hydroxyproline in tilapia skin is ASC (70 residues) and PSC (86 residues). As a characteristic component of collagen, the content of collagen in raw materials can be measured by the ratio of hydroxyproline. No cystine and tryptophan were detected in both the ASC and PSC of tilapia skin collagen, which was consistent with the characteristics of type I collagen.

### 2.3. FTIR Analysis

**Figure 1:**
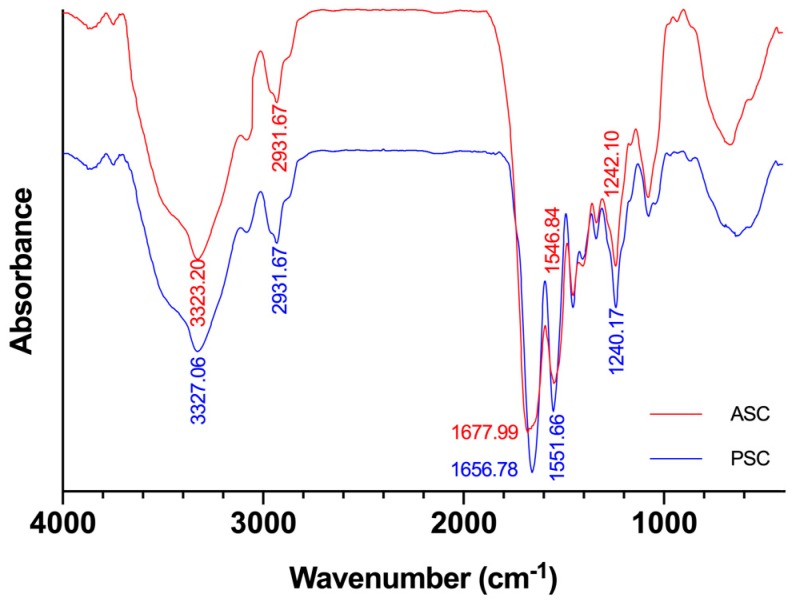
Fourier transform infrared spectroscopy (FTIR) of ASC and PSC from Nile tilapia skin.

The FTIR spectra of ASC and PSC are exhibited in Figure 1. Each peak in the FTIR spectrums corresponds to the vibration of functional groups in the molecule [55]. The secondary structure of collagen is closely related to different types of hydrogen bonds [56]. By analyzing the FTIR spectrum of ASC and PSC, the different effects of the two extraction methods on the secondary structure of collagen were obtained. At room temperature, ASC and PSC mainly exhibited five absorption peaks at 3323 cm^−1^, 2931 cm^−1^, 1677 cm^−1^, 1546 cm^−1^, and 1242 cm^−1^, corresponding to amide A, amide B, amide I, amide II, and amide III, respectively.

The wavenumber of the free N–H stretching vibration was located in the range of 3400–3440 cm^−1^, and the wavenumber of amide A was measured at 3323 cm^−1^, indicating that the N–H stretching vibration and the hydrogen bonding are combined [51]. The amide A absorption peak of PSC at 3323.20 cm^−1^ is slightly higher than ASC at the wavenumber of 3327.06 cm^−1^, indicating that more N–H groups in the ASC are hydrogen-bonded, which suggested that the PSC is slightly weaker than ASC in structural stability. Both ASC and PSC have a weak absorption peak at the amide B band at 2931.67 cm^−1^, indicating the asymmetric stretching vibration of –CH_2_ [57]. Studies have shown that the amide I, amide II, and amide III bands are related to the triple-helix structure of collagen [58]. The amide I bands were attributed to C=O stretching vibration, and the amide I absorption bands of ASC and PSC appeared at 1677.99 cm^−1^ and 1654.78 cm^−1^, respectively. The red shift of C=O stretching vibration may be caused by the use of the pepsin-degraded part of the telopeptides during the preparation process. The telopeptides plays an important role in the triple-helix structure of collagen, which was attributed to the covalent aldol cross-linking and the collagen fiber formation. Excision of the telopeptides does not completely destroy the natural collagen structure [59], but it leads to an incomplete collagen protein structure and increased solubility [60], which explained why the PSC yields are higher than those of the ASC. From the consideration of biomedical materials applications, much attention should be paid to the effects of the telopeptides on the immunogenicity of collagen. It has been reported that the immunogenicity of collagen’s telopeptides was considered the most important factor of the collagen-induced immune response [59]. Previous studies have also suggested that the peptides located at the center of the triple helix of pepsin-treated collagen (from skin of bovine) are the major antigenic sites that cause human immune responses [61].

Amide II bands produced by N–H bending vibrations and C–N stretching vibrations are usually located in the range of 1550 to 1600 cm^−1^. Research has shown that the red shift of the amide II peak is related to the hydrogen bond increase of the N–H group [39]. The amide II absorption bands of ASC and PSC were detected at wavenumbers of 1546.84 cm^−1^ and 1551.66 cm^−1^, respectively. The result indicates that there are more hydrogen bonds between the peptide chains in ASC than PSC.

The amide III bands represent the combination of the C–N stretching vibration and the N–H bending vibration [56]. The amide III absorption bands of ASC and PSC appeared at 1242.10 cm^−1^ and 1240.17 cm^−1^ respectively, which is consistent with previous studies [41].

### 2.4. Thermal Denaturation Temperatures of ASC and PSC

**Figure 2:**
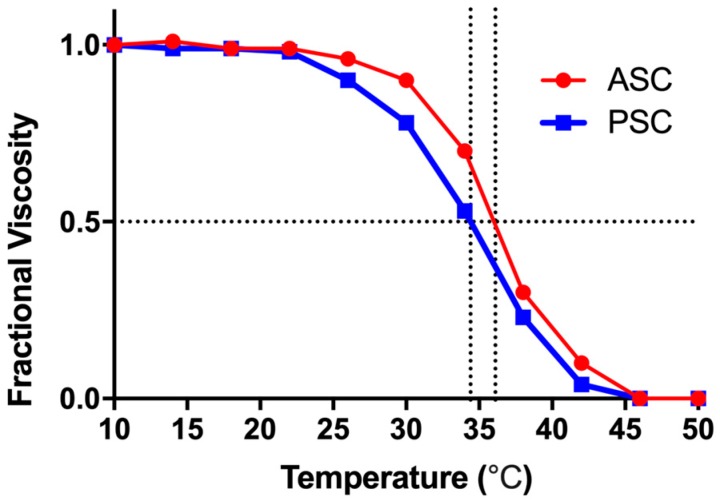
Thermal denaturation curves of ASC and PSC from Nile tilapia skin. The denaturation temperature was determined as the mid-point temperature where viscosity changes reach 0.5.

The tripeptide chains are bound by non-covalent bonds such as hydrogen bonds, which is the basis of the stability of collagen. When the collagen molecules absorbed enough heat from the outside, these non-covalent bonds were destroyed, causing the triple-helix structure to become a random coil structure and destroying the biological properties of collagen. The thermal stability of ASC and PSC were studied by viscosity measurement [62]. According to Figure 2, ASC and PSC have similar curves, and their denaturation temperatures (Td) were 36.1 °C and 34.4 °C, respectively. The Td values can be regarded as the temperature at which the triple-helix structure of collagen is deformed into a random coil structure. The Td values of ASC and PSC from tilapia skin are similar to the collagen extracted from fish living in warm tropical climates such as salmon (29.3 °C) and bigeye snapper (30.4 °C) [49], and higher than the cold-water fish, such as Baltic herring (15.0 °C) and Argentine salmon (10.0 °C) [63], but lower than terrestrial animals such as bovine (39.7 °C) or porcine (37 °C) [64]. The difference in amino acid composition was the primary cause of the different thermostability of collagen. The loss of the PSC telopeptides has a certain influence on the stability of the triple-helix structure, resulting in lower thermal stability than ASC, which is consistent with the results of amino acid composition analysis and FTIR analysis. Although the thermal stability of tilapia skin collagen is lower than that of terrestrial organisms, it is higher than that of common aquatic organisms, which is an advantage for its application in the field of biomedical materials.

### 2.5. SDS-PAGE

**Figure 3:**
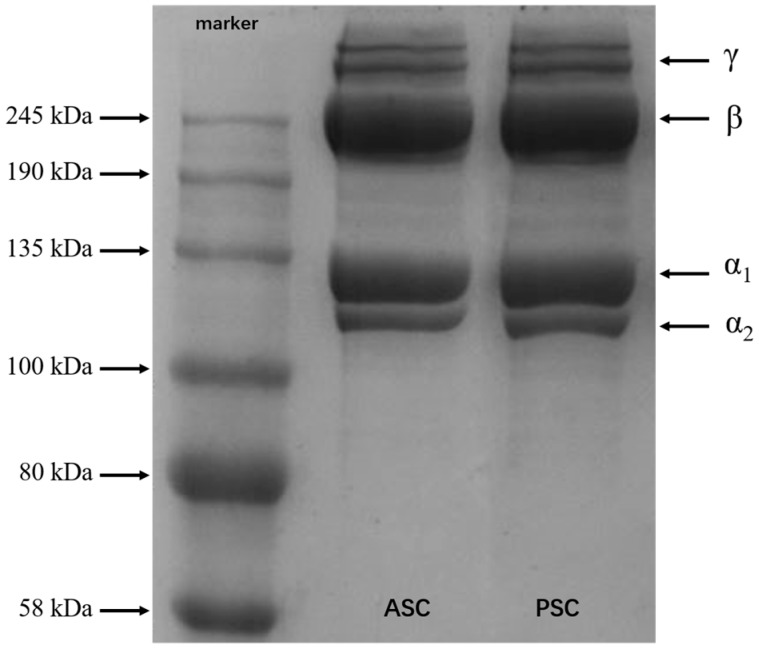
SDS-PAGE patterns of ASC and PSC from Nile tilapia skin.

SDS-PAGE results present the subunit composition and type of collagen instinctively. As shown in Figure 3, ASC and PSC have very similar protein bands, which have been identified as trimers (γ chains), dimers (β chains), and two alpha chains (α_1_ and α_2_). Up to now, it has been reported that there are two different collagen trimers in tilapia skin, (α_1_)_2_α_2_ and α_1_α_2_α_3_, and that the two chains of α_1_ and α_2_ have the same molecular weight, which cannot be distinguished by electrophoresis [65]. According to Sun [41], the structure of Nile tilapia collagen is (α_1_)_2_α_2_ type. The band intensity of α_1_ is twice that of α_2_, indicating that both ASC and PSC are type I collagen. In addition, the electrophoresis bands are clear, and have no low molecular weight bands, showing that the molecular structure of collagen was well preserved during the extraction process. Although some telopeptides were lost after the treatment of PSC, the banding pattern of PSC was similar to ASC, indicating that the PSC extraction process does not affect the integrity of the triple-helix structure.

### 2.6. Morphology

**Figure 4:**
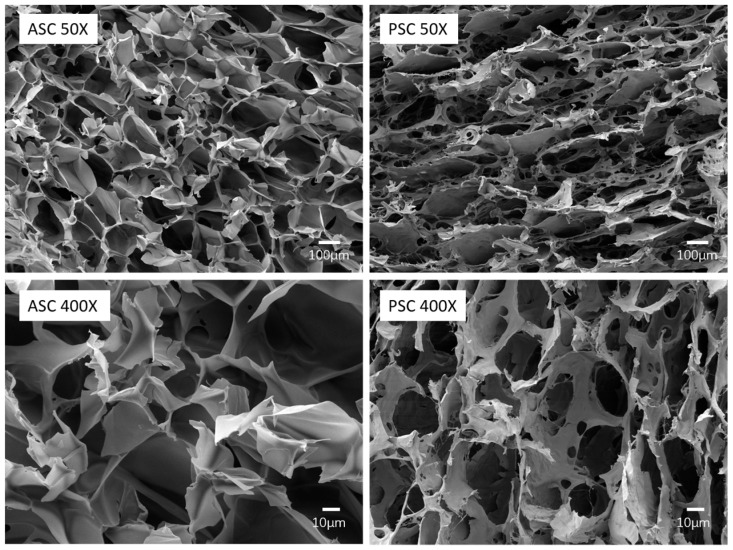
Morphological features of Nile tilapia skin collagen using SEM.

Figure 4 shows the ultrastructure of the cross-section of the lyophilized collagen sponge. The graphs showed that ASC and PSC exhibit slightly different microscopic morphology. From the 50× magnified image, ASC and PSC display a loose porous network structure, but the pores of ASC show a more uniform and less fiber structure pattern than PSC. As shown in the 400× magnified image, ASC is a dense sheet-like film with uniform alignment, and PSC exhibits irregularly arranged curls.

The microstructures determine the physicochemical properties and biofunctionality of the materials; they have a significant value to the application of collagen in biomedical materials. The SEM results of ASC present typical characteristics of aquatic collagen, such as miiuy croaker [56] and Acipenser schrenckii [66], while the microstructures of PSC exhibit a fibrous structure similar to that of terrestrial collagen, such as bovine [67]. This difference may be caused by the structure of PSC being changed under the influence of pepsin, forming more collagen fibers in a non-crosslinked state, which is consistent with the results of FTIR. In the field of biomedical materials, greater porosity facilitates the migration of cells into the interior of the scaffolds, which has certain advantages for promoting wound healing. The lower degree of cross-linking is beneficial to the dissolution and re-processing of collagen, and is suitable for many processes such as electrospinning. Therefore, the microstructures of ASC and PSC indicate that they are appropriate for use in different biomedical material fields.

### 2.7. Endotoxin Test

Endotoxin, which is also as known as lipopolysaccharide, is found in the outer membrane of Gram-negative bacteria. When endotoxin invades the body, it can cause shock, fever, a fall in blood pressure, and death [68,69]. Endotoxin must be eliminated as much as possible from biomedically used materials. We used an endotoxin denial test to investigate the biosafety of ASC and PSC. The results show that negative results were obtained in endotoxin (below 0.01 EU/mL) in leach liquor of ASC and PSC.

### 2.8. Cell Proliferation

**Figure 5:**
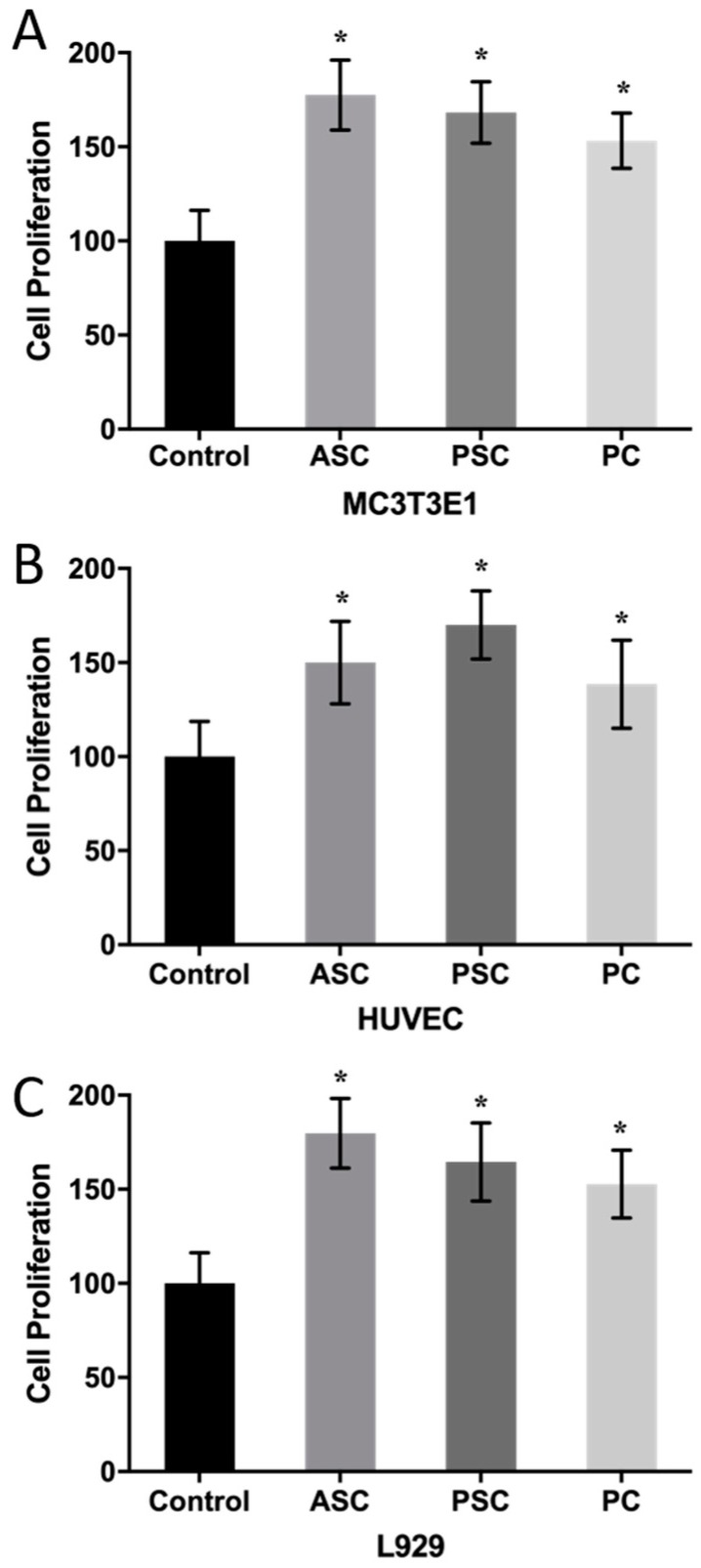
The effect of ASC, PSC, and porcine collagen (PC) on cell proliferation. (**A**): MC3T3E1, (**B**): L929, (**C**): HUVEC. Values with * show significant differences (*p* < 0.05) between groups, as determined by one-way ANOVA.

In order to fully reflect the application potential of ASC and PSC in biomedical materials, MC3T3E1, L929, and human umbilical vein endothelial cells (HUVEC) cells were selected to test its in vitro cytotoxicity. Porcine collagen (PC) was used as a comparison group. According to Figure 5, the addition of collagen significantly promoted cell proliferation compared to the control group. MC3T3E1 and L929 cells had higher proliferation rates in the ASC treatment groups. However, HUVEC had a higher proliferation rate after PSC treatment. Besides, the ASC and PSC treatment groups had higher proliferation rates than the PC group in all three kinds of cells. It has been reported that Nile tilapia collagen contains an antibacterial tilapia piscidin (tp4) that has the ability to stimulate cell proliferation and activate epidermal growth factor (EGF), transforming growth factor (TGF), and vascular endothelial growth factor (VEGF) [70]. In the study of blue shark skin collagen, PSC had a higher proliferation rate for differentiated mouse bone marrow-mesenchymal stem (dMBMS) cells than ASC, while ASC and PSC had no significant difference regarding the proliferation rate of MC3T3E1 cells [71]. The results indicate that both ASC and PSC have potential application in fields requiring wound dressing. The osteogenic properties of ASC make it suitable for applications in the field of bone and cartilage repair, while PSC could be used in areas such as the promotion of angiogenesis or artificial blood vessels.

### 2.9. Biocompatibility Evaluation

**Figure 6:**
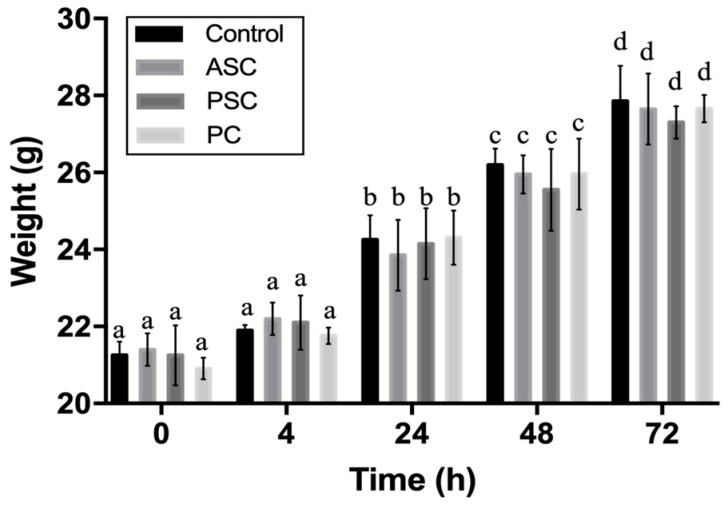
Weight changes of mice after intraperitoneal injection. The different letters in the same group (same type of the bar) represent significant difference (*p* < 0.05).

The acute systemic toxicity assay was usually selected to measure the adverse effect of biomedical materials that result either from a single exposure or from multiple exposures in a short period of time. It is an important indicator of biosafety assessment [72]. To evaluate the biosafety of aquatic collagen, porcine collagen was designated as a comparison group. As shown in Figure 6, no significant difference in body weight was observed between the experimental groups and the control group at four hours, 24 h, 48 h, and 72 h after the intraperitoneal injection of the collagen leach liquor. Moreover, the weight of the control group and the experimental groups increased significantly over time, and no dead samples appeared. Furthermore, there were no significant differences between the ASC, PSC, and PC groups. The results showed that ASC and PSC collagen sponges were produced by our process without acute systemic toxicity, and there was not a significant difference from the acute toxicity from commercially available porcine collagen products.

## 3. Materials and Methods

### 3.1. Raw Materials

Nile tilapia skins were procured from Zhenhua Aquatic Product Company (Guangzhou, Guangdong province of China). Frozen skins were thawed with running water and removed from the residuals manually. They were chopped into small pieces and stored at −20 °C. Porcine collagen (PC) was obtained from Kele Biotech (Chengdu, China). All of the other reagents used were of analytical grade.

### 3.2. Preparation of ASC

All the procedures were carried out at 4 °C to minimize collagen denaturation. An acellular environment was used in the extraction process to reduce the exogenous pyrogen. The pieces of Nile tilapia skins were soaked with 0.1 M of NaOH for 24 h with continuous stirring to remove the non-collagenous proteins. Washing the fish skins repeatedly with cooled deionized water ensured that they were neutralized. Adding 0.5 M of acetic acid in a ratio of 1:50 (*w*/*v*) started extraction for two days. Then, they were centrifuged at 10,000 rpm for 30 min at 4 °C. NaCl was added to the collected supernatants until a concentration of 0.9 M was reached to salt out the collagen. The precipitated collagen was separated by centrifugation at 10,000 rpm for 30 min at 4 °C and dissolved in 0.5 M of acetic acid. Then, the solution was dialyzed for 24 h against 0.1 M of acetic acid in a dialysis membrane with a molecular weight cut-off of 50 kDa, and then for 48 h against ultra-pure water; the water was changed every eight hours. The resulting collagen was freeze-dried for three days and sealed in polythene bags until further use.

### 3.3. Preparation of PSC

The extraction process of PSC was basically identical to the extraction of ASC except for slightly differences. The tilapia skins were extracted by 0.5 M of acetic acid containing 0.1% (*w*/*v*) pepsin for 48 h with stirring. Then, the supernatant was dialyzed against 0.02 M of Na_2_HPO_4_ for 24 h with solution changed every eight hours before being dialyzed against 0.1 M of acetic acid.

### 3.4. Extraction Yield of ASC and PSC

The calculation of ASC and PSC yield referred to previous reports [73] and the equation was as follows:

yield (%) = weight of dried collagen (g) × 100/weight of dried skins (g)

### 3.5. Amino Acid Composition

The ASC and PSC samples were hydrolyzed by dissolving in 6 M of HCl at 110 °C for 24 h. The solution was analyzed with an amino acid analyzer (835-50, Hitachi, Tokyo, Japan).

### 3.6. Denaturation Temperature (Td)

The denaturation temperature of collagen from tilapia skin was determined by differential scanning calorimetry (DSC) (Netzsch DSC 200PC, Selb, Bavaria, Germany). A collagen sample with a concentration of 5 mg/mL was dissolved in 0.05 M of acetic acid and sealed in an aluminum pan for scanning. Then, the endothermal curve from 10 °C to 50 °C was obtained at a rate of 5 °C/min in a nitrogen atmosphere.

### 3.7. Fourier Transform Infrared Spectroscopy (FTIR)

The infrared absorption characteristics of collagen were obtained by an FTIR spectrometer (Tensor 27, BRUKER, Bremen, Germany). First, one-mg collagen samples were mixed with potassium bromide (KBr) at a ratio of 1:100 and pressed into pellets with a manual mechanical squeezing device. The spectra were recorded with a wavenumber range from 4000 to 400 cm^−1^ at the resolution of 2 cm^−1^.

### 3.8. Sodium Dodecyl Sulphate-Polyacrylamide Gel Electrophoresis (SDS-PAGE)

The protein molecular mass analysis was studied by SDS-PAGE according to Laemmli [74] with 7.5% resolving gel and 5% stacking gel and 120 voltage using a Bio-Rad electrophoresis. The protein bands were stained with Coomassie Blue R250 and destained with 30% (*v*/*v*) methanol and 10% (*v*/*v*) acetic acid. The molecular mass of the subunits was analyzed by the location of the bands.

### 3.9. Scanning Electron Microscopy (SEM)

The morphology of ASC and PSC was observed using a scanning electron microscope (JSM-840, JEOL, Tokyo, Japan) operated at 5 kV. The samples were pasted on a blade and sputter-coated with gold at 30 mA for 15 min. The SEM images were obtained at 50× and 400× magnification.

### 3.10. Endotoxin Denial Test

An endotoxin denial test was undertaken using a chromogenic end-point tachypleus amebocyte lysate (CE TAL) assay kit (Beijing Solarbio Science & Technology Co., Ltd., Beijing, China). Test samples and standards were prepared according to manufacturer’s guidelines. Absorbance was measured at 405 nm using a microplate spectrophotometer (SynergyTM 2, BioTek, Winooski, VT, USA). Data were corrected to exclude background readings. A negative result was defined as a level below 0.01 EU/mL [75].

### 3.11. Cell Culture

MC3T3E1 and L929 cells were obtained from a cell bank (Procell Life Science & Technology Co., Ltd., Wuhan, China) and HUVEC were received from an institute of zoology (Chinese academy of sciences, Beijing, China). MC3T3E1 was grown as monolayer at 37 °C in a 5% CO_2_ incubator and cultured in Dulbecco’s modified eagle medium (DMEM) containing 10% (*v*/*v*) fetal bovine serum (FBS) and 1% (*w*/*v*) penicillin-streptomycin. L929 and HUVEC cells were cultured in MEM and DMEM/F12 (1:1), respectively.

### 3.12. Cytotoxicity In Vitro

The collagen samples were cut into disks sized for a 48-well plate and sterilized by ^60^Co for 18 h with an accumulative dose of 20 kGy before using. The cells were seeded a concentration of 1 × 10^4^ cells/well and cultured with a specific medium. After seeding for 48 h, 50 μL of thiazolyl blue tetrazolium bromide (MTT) solution was added to the wells and incubated for four hours at 37 °C/5% CO_2_. Wells with collagen were used as negative controls of cytotoxicity. We dissolved the formazan salts using DMSO with shaking for 10 min and replaced the solution with blank wells. The absorbance was measured using a 96-well microplate reader at 490 nm [76].

### 3.13. Acute Systemic Toxicity Assay

All animal procedures were approved by the ethical committee of animal research in the Ocean University of China, and complied with the requirements of the National Act on the use of experimental animals (China). The acute systemic toxicity of tilapia collagen was investigated according to ISO 10993-11:2009. The collagen samples were cut into 1×1 cm sizes with 0.5-mm thickness and sterilized by ^60^Co before using. The leach liquor was prepared at a ratio of three cm^2^/mL collagen into saline between the total surface area of the materials for 72 h at 37 °C. Then, 20 Kunming (KM) mice (Licensed ID: SCXK 2014-0007) were divided into four groups randomly. Three experimental groups were intraperitoneally injected the leach liquor of ASC, PSC, and PC with a dose of 50 mL/kg respectively, and the blank control group was injected saline with a dose of 50 mL. The weight of mouse was weighed and recorded immediately after injection and at four hours, 24 h, 48 h, and 72 h after injection. The criteria that were used to assess acute systemic toxicity are shown in Table 2.

### 3.14. Statistical Analysis

All of the experiments were replicated in triplicate and the values were expressed as means ± standard deviation (SD). The analyses of multiple groups by one-way ANOVA were using Macintosh GraphPad Prism Version 7, and we considered p-values of less than 0.05 to measure the significant differences.

## 4. Conclusions

This study showed that ASC and PSC extracted from Nile tilapia skin have typical type I collagen characteristics. No significant differences were found in the amino acid composition and physicochemical properties of ASC and PSC. Both ASC and PSC have a complete triple-helix structure. The thermal stability of PSC was slightly lower than that of ASC, similar to mammals, and higher than cold-water fish. ASC has the effect of promoting osteogenesis and fibroblastation, while PSC is beneficial to the formation of vascular endothelial cells by comparison, and neither ASC nor PSC cause acute systemic toxicity. In summary, collagen extracted from Nile tilapia skin is a biocompatible type I collagen with potential as a biomedical material.

## Figures and Tables

**Table 1:** The amino acid composition of acid-soluble collagen (ASC) and pepsin-soluble collagen (PSC) from Nile tilapia skin.

Amino acid	PSC	ASC
Hydroxyproline	70	86
Aspartic acid	37	40
Threonine	17	15
Serine	28	31
Glutamic acid	93	98
Proline	115	106
Glycine	343	322
Alanine	85	87
Valine	25	22
Methionine	10	9
Isoleucine	16	11
Leucine	20	22
Tyrosine	6	9
Phenylalanine	16	10
Lysine	22	32
Histidine	12	10
Arginine	85	90
Total	1000	1000

**Table 2:** The criteria used to assess acute systemic toxicity.

Acute Systemic Toxicity	Conditions after Treatment with the Test Sample
Negative (−)	None of the five animals showed a significantly greater biological reactivity.
Positive (+)	Two or more of the five animals died.
	Two or more of the five animals showed behavior such as convulsions or prostration.
	Three or more of the five animals showed a body weight loss greater than 10%.
